# A machine learning model for predicting oligoclonal band positivity using routine cerebrospinal fluid and serum biochemical markers

**DOI:** 10.1093/ajcp/aqaf119

**Published:** 2025-12-06

**Authors:** Hazar Gözgöz, Oğuzhan Orhan, Başak Akan Konuk, Pınar Akan

**Affiliations:** Faculty of Medicine, Department of Clinical Biochemistry, Dokuz Eylul University, Izmir, Turkey; Engineering Faculty, Computer Engineering, Izmir Institute of Technology, Izmir, Turkey; Computation, Information and Technology (CIT), Technical University of Munich, Munich, Germany; Faculty of Medicine, Department of Clinical Biochemistry, Dokuz Eylul University, Izmir, Turkey

**Keywords:** oligoclonal bands, cerebrospinal fluid, machine learning, multiple sclerosis, diagnostic prediction model

## Abstract

**Objective:**

To develop and validate a machine learning model for predicting oligoclonal band (OCB) positivity using routine cerebrospinal fluid (CSF) and serum biochemical markers to improve the diagnostic accuracy and efficiency of assessing intrathecal immunoglobulin G (IgG) synthesis.

**Methods:**

In this retrospective study (n = 1709), an ensemble model was developed using 8 refined CSF and serum parameters. Combining optimized CatBoost, XGBoost, and LightGBM classifiers, the model was trained and evaluated using a 2-phase workflow, including 5-fold cross-validation and validation on independent internal (n = 342) and external (n = 49) cohorts.

**Results:**

The developed ensemble model achieved a receiver operating characteristic–area under the curve (ROC-AUC) of 0.902 on the internal test set, significantly outperforming the conventional IgG index (ROC-AUC, 0.795). At its optimal threshold, the model demonstrated an accuracy of 0.830, with a sensitivity of 0.714 and a specificity of 0.916. On the external validation cohort, it achieved 90% accuracy and 96% sensitivity.

**Conclusions:**

A novel machine learning ensemble model accurately predicts OCB positivity using routine laboratory data and demonstrates superior performance compared with the IgG index. This approach represents a significant step in applying artificial intelligence in laboratory medicine, with the potential to enhance diagnostic efficiency. Prospective, multicenter validation is essential for broader clinical implementation.

KEY POINTS

- This study addresses the need for a more objective tool to supplement oligoclonal band (OCB) analysis in cerebrospinal fluid.
- A novel machine learning model using routine laboratory data accurately predicts OCB positivity, outperforming the conventional immunoglobulin G index.
- This model can serve as a decision support tool to enhance diagnostic efficiency and workflow in clinical laboratories.

## INTRODUCTION

Cerebrospinal fluid (CSF) analysis is a cornerstone in the diagnosis of neurologic diseases, providing critical insights into the pathologic processes of the central nervous system (CNS).^1^ A key finding from this analysis is the presence of 2 or more CSF-restricted oligoclonal bands (OCBs), which indicates intrathecal immunoglobulin G (IgG) synthesis and is considered the most sensitive and reliable laboratory marker for multiple sclerosis (MS),^2^ a chronic autoimmune disease characterized by demyelination and axonal injury and the most common nontraumatic cause of neurologic disability in young adults.^3^^,^^4^ Analysis of CSF remains vital for MS diagnosis, especially in atypical or ambiguous clinical and radiologic presentations.^4^ However, the clinical significance of OCBs extends beyond MS, as they may also be found in other CNS inflammatory, infectious, and systemic diseases.^5^^,^^6^

The current gold-standard method for OCB detection is isoelectric focusing (IEF) with subsequent IgG-specific immunoblotting.^7^ Despite its high sensitivity, this technique has several inherent limitations. The interpretation of OCB patterns is often subjective, leading to interobserver variability; it is also labor-intensive and has a long turnaround time.

Quantitative indices, such as the IgG index, which compares CSF and serum IgG and albumin concentrations, provide supplementary diagnostic value, although their sensitivity is lower than that of OCB analysis.^2^^,^^8^ Current diagnostic guidelines prioritize OCB detection, while acknowledging the utility of the IgG index in routine practice due to its rapid availability.^2^ This represents an ongoing challenge in laboratory medicine: balancing the high sensitivity of OCB detection with its subjectivity and labor-intensive workflow, while considering the rapid availability but lower sensitivity of the IgG index.

This diagnostic gap, alongside the growing need to improve diagnostic timeliness and confidence and the increased risk of misdiagnosis in atypical cases,^4^ highlights the need for novel analytical approaches to enhance diagnostic accuracy. Machine learning (ML) offers a robust framework for modeling the complex relationships between routinely available quantitative CSF and serum parameters to predict the qualitative OCB result.

In this study, we present an ML-based prediction model that integrates these quantitative parameters to predict OCB positivity. We hypothesized that an ML-supported approach could serve as a valuable decision support tool to complement the current diagnostic workflow, potentially improving the diagnostic objectivity and efficiency of detecting intrathecal IgG synthesis in diagnostically challenging cases.

## MATERIALS AND METHODS

### Study design and participants

This retrospective cross-sectional study included patients aged 18 to 90 years who presented to Dokuz Eylul University Hospital between January 1, 2006, and December 15, 2022, and had requests for both OCB analysis and IgG index testing for whom these tests were performed as part of routine clinical care.

The CSF and serum samples collected for these analyses were routinely analyzed in the Dokuz Eylul University Hospital Central Laboratory.

The study utilized routinely ordered OCB images, CSF IgG, CSF albumin, CSF total protein, CSF glucose, CSF sodium, CSF potassium, CSF chloride, CSF lactic acid, CSF microalbumin, serum IgG, serum albumin, and serum C-reactive protein (CRP) levels, as well as CSF white blood cell (WBC) and red blood cell (RBC) counts and erythrocyte sedimentation rate (ESR) results.

Samples with unsuitable preanalytical or analytical conditions, including hemolysis, lipemia, icterus, or invalid measurements, were excluded. Samples with compromised biochemical integrity due to improper temperature storage or delayed delivery were also excluded. Results considered invalid, such as those with obvious measurement errors or patient identification confusion, were not included in the study.

The final analytical cohort was restricted to cases for which the complete panel of analytes used for model development was available. These criteria were defined to ensure preanalytical and analytical standardization and to maximize the accuracy of the collected data.

The study was conducted in accordance with the Declaration of Helsinki and received approval from the Dokuz Eylul University Non-Invasive Research Ethics Committee on June 14, 2023 (decision number: 2023/20-07). Due to the retrospective nature of the study, the requirement for individual patient consent was waived by the committee.

### Sample collection and laboratory measurements

All laboratory analyses were performed according to standardized protocols in the Dokuz Eylul University Hospital Central Laboratory. The analytical performance characteristics of the employed assays, reflecting validated in-house performance, are detailed below.

### Nephelometric assays

Quantitative concentrations of immunoglobulins and proteins were determined by nephelometry on the Atellica NEPH 630 analyzer (Siemens Healthineers).

### Clinical chemistry assays

All chemistry parameters were measured on the AU5800 chemistry analyzer (Beckman Colter). CSF total protein was measured using the pyrogallol red molybdate method. CSF glucose was measured enzymatically via the hexokinase method. CSF sodium, CSF potassium, and CSF chloride were analyzed using an ion-selective electrode module. CSF lactic acid was measured photometrically, and serum CRP was measured using an immunoturbidimetric method.

### Hematology and manual assays

ESR was determined from whole blood using the Test 1 system (Alifax). CSF WBC and RBC counts were performed manually using a Neubauer counting chamber.

### OCB membrane results classification

The current gold-standard method involves IEF, followed by IgG-­specific immunoblotting. This methodology identifies 5 distinct immunofixation patterns by analyzing paired CSF and serum samples, as recommended by international consensus. These patterns include type 1, representing a normal finding with no bands in either CSF or serum; type 2, indicating intrathecal IgG synthesis with OCBs present only in the CSF; type 3, showing OCBs in the CSF along with identical oligoclonal bands in both fluids; type 4, characterized by identical bands in both CSF and serum without any CSF-restricted bands, indicative of a systemic immune reaction; and type 5, which shows monoclonal bands in both fluids.^2^ For developing the ML model, a binary classification was used. Samples exhibiting type 2 or type 3 patterns were classified as OCB-positive, as both indicate the presence of intrathecal IgG synthesis. All other patterns (types 1, 4, and 5) were classified as OCB-negative.

### OCB interpretation and pattern distribution

All IEF results were independently interpreted by 2 experienced laboratory specialists, P. A. and H. G. . In cases of disagreement, the immunoblot was reexamined by a third senior specialist to reach a consensus. The distribution of OCB patterns in the final cohort of 1709 cases was as follows: type 1 (n = 184, 11%), type 2 (n = 116, 7%), type 3 (n = 606, 35%), type 4 (n = 801, 47%), and type 5 (n = 2, <1%).

### Machine learning workflow

**Figure 1:**
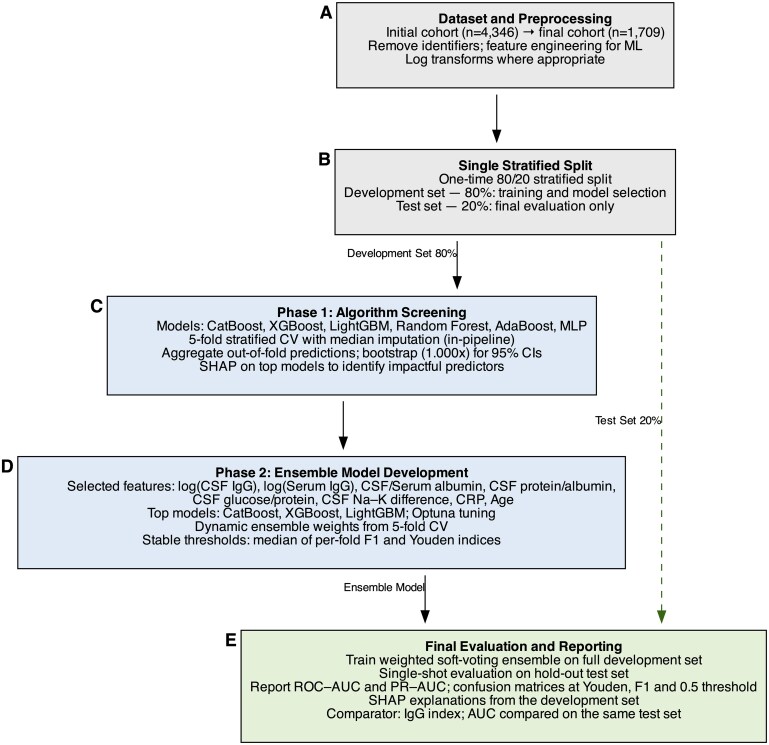
Schematic overview of the 2-phase machine learning workflow. **A**, The workflow begins with data set preprocessing and feature engineering. **B**, The full cohort is then partitioned into a development set (80%) and a sequestered hold-out test set (20%). **C**, In phase 1, multiple algorithms are screened on the development set using 5-fold cross-validation to identify the most promising model family and the most impactful features via SHAP analysis. **D**, In phase 2, the insights from the screening are used to select, optimize, and construct a final ensemble model. **E**, Finally, the optimized model is evaluated for the first and only evaluation on the unseen hold-out test set to ensure an unbiased assessment of its performance. CSF, cerebrospinal fluid; CV, cross-validation; ML, machine learning; MLP, multilayer perceptron; PR-AUC, precision-recall–area under the curve; RF, random forest; ROC-AUC, receiver operating characteristic–area under the curve; SHAP, SHapley Additive exPlanations.

The ML analysis was developed following a structured workflow, adhering to the principles recommended by the International Federation of Clinical Chemistry and Laboratory Medicine Working Group on Machine Learning for developing robust and clinically applicable models.^9^ The systematic, 5-phase process, as illustrated in Figure 1, was designed to first perform a comprehensive screening of multiple algorithms, followed by the development and final validation of a specialized ensemble model. The Python code used for the analysis is available at https://github.com/HazarGozgoz/ocb_prediction_ml.

### Data set preparation and splitting

**Figure 2:**
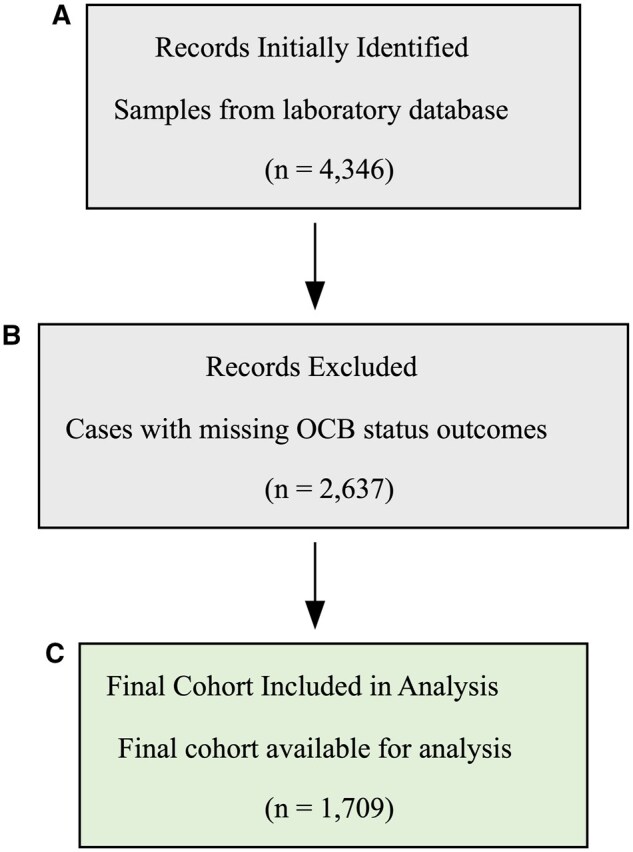
Flowchart of the study cohort selection process. The diagram illustrates the sequential steps for patient inclusion: (**A**) identification of initial records from the laboratory database, (**B**) exclusion of records with missing oligoclonal band status outcomes, and (**C**) the resulting final cohort for analysis.

A total of 4346 CSF and serum sample results were initially considered. After programmatically removing all patient identifier columns and excluding cases with missing OCB status outcomes, a final cohort of 1709 cases was established for analysis. Most of these exclusions were due to a test not being requested, with a small fraction (approximately 2%) being due to technically uninterpretable results, such as those with poor protein transfer to the membrane, high background staining, or blurry bands that precluded a definitive interpretation. The patient selection process is detailed in Figure 2. This exclusion was primarily due to cases where OCB testing was not clinically requested or where results were technically uninterpretable. To ensure a final, unbiased evaluation, the full data set (n = 1709) was then stratified by OCB status and partitioned into a development set (n = 1367, 80%) and an independent hold-out test set (n = 342, 20%). All subsequent model development was performed exclusively on the development set to prevent data leakage. Additionally, descriptive analysis revealed significant positive skew in the distributions of CSF IgG (skewness = 5.19; Figure S1A) and serum IgG (skewness = 0.72; Figure S1B), prompting the use of log-transformed values (log[1 + x]) in model development. Demographic characteristics (age and sex) were well balanced between the development and test sets, as shown in Table S1.

### Feature engineering and preprocessing

To enrich the feature set, several new variables were engineered, including key clinical ratios (CSF/serum albumin ratio, CSF glucose/protein ratio, CSF sodium-potassium difference) and logarithmic transformations (log[1 + x]) of immunoglobulin concentrations. The comprehensive feature set used for the initial model screening consisted of 24 features. All cross-validation workflows were implemented via the scikit-learn Pipeline, including median imputation (SimpleImputer) fitted within each training model fold to prevent data leakage. In the final cohort, no predictor had missingness; thus, imputation did not alter values but preserved a leakage-safe, reproducible pipeline.

## PHASE 1: COMPREHENSIVE ALGORITHM SCREENING

Six models (random forest, AdaBoost, multilayer perceptron [MLP], XGBoost, LightGBM, and CatBoost) were evaluated with default hyperparameters using 5-fold stratified cross-validation (shuffle = True, random_state = 42). Performance was summarized on out-of-fold predictions with the nonparametric bootstrap (1000 iterations) to derive 95% CIs for receiver operating characteristic (ROC)–area under the curve (AUC), precision-recall (PR)–AUC, accuracy, sensitivity, specificity, precision, and F1. SHapley Additive exPlanations (SHAP) analyses on the top performers were used to identify the most influential predictors.

## PHASE 2: FINAL ENSEMBLE MODEL DEVELOPMENT

Guided by phase 1, we selected an 8-feature panel: log-transformed CSF IgG, age, CSF protein/albumin ratio, log-transformed serum IgG, CSF ­sodium-potassium difference, CRP, CSF glucose/protein ratio, and CSF/serum albumin ratio. The top 3 models (CatBoost, XGBoost, and LightGBM) underwent hyperparameter tuning with Optuna (50 trials per model), maximizing the mean ROC-AUC across 5-fold cross-validation on the development set. Subsequently, a final weighted soft-voting ensemble model, designated SYNAPSI (Synthesis Analysis Prediction System for IgG), was constructed. The weights for combining the 3 optimized models were assigned proportionally to each model’s mean cross-validation ROC-AUC. To ensure robust classification, stable decision thresholds were established by computing the F1-optimal and Youden index (J) optimal thresholds in each cross-validation fold on the development set, with the median of these values used as the final thresholds.

## PHASE 3: FINAL EVALUATION AND INTERPRETABILITY

The optimized ensemble was trained on the entire development set and evaluated once on the untouched hold-out test set. Overall performance was assessed using ROC-AUC and PR-AUC, while threshold-dependent metrics were reported at 3 distinct decision thresholds: the F1 score–maximizing threshold, the Youden index (J)–optimal threshold, and the conventional 0.5 threshold. Model calibration was assessed with 10-bin reliability curves (Figure S2). To benchmark against the conventional IgG index, we computed the index as


$$\mathrm{IgG}\;\mathrm{index}=\mathrm{CSF}\frac{\mathrm{IgG}}{\mathrm{Albumin}}\times \mathrm{Serum}\frac{\mathrm{Albumin}}{\mathrm{IgG}}$$

and evaluated its AUC on the same test set using the conventional 0.70 cutoff for threshold metrics. The AUCs were compared using an individual-level paired bootstrap (2000 iterations) with 95% CIs and 2-tailed *P* value and was complemented by a paired DeLong test.

### External validation

To obtain preliminary evidence of the model’s generalizability, an independent external cohort of consecutive samples was used. These samples were analyzed in our laboratory during a subsequent time period not included in the main data set, providing a measure of temporal validation. To ensure consistency in the gold-standard labeling, OCB interpretation for this cohort was performed by the same 3 laboratory specialists who evaluated the primary data set.

### Statistical analysis

Descriptive statistical analyses were performed using IBM SPSS Statistics for Mac (version 29.0; IBM Corp). Due to nonnormal distributions confirmed by the Shapiro-Wilk test, continuous variables were summarized as medians and IQRs (25th-75th percentiles).

All ML workflows were developed and executed in Python (version 3.11). The primary libraries used included scikit-learn for ML pipelines and ­metrics; pandas for data management; XGBoost, CatBoost, and LightGBM for gradient boosting models; Optuna for hyperparameter optimization; and SHAP for model interpretability. A 2-sided *P* value of less than .05 was considered statistically significant for all analyses.

## RESULTS

### Characteristics of the study cohort

After applying the exclusion criteria, a final cohort of 1709 patients was included in the analysis, of whom 987 (58%) were OCB-negative and 722 (42%) were OCB-positive. The median age of the cohort was 42.0 years (IQR, 35.0-55.0), and it comprised 988 (58%) men and 721 (42%) women. A detailed age group distribution is provided in Table 1.

**Table 1:** Demographic Characteristics of the Study Population^a^

Variable	Category	No. (%)
**Sex**	Female	721 (42)
	Male	988 (58)
**Age group, y**	18-30	252 (15)
	31-40	572 (34)
	41-50	304 (18)
	51-60	276 (16)
	61-70	219 (13)
	71-80	63 (4)
	81-90	23 (1)
**OCB status**	Negative	987 (58)
	Positive	722 (42)

Abbreviation: OCB, oligoclonal band.

a Percentages are rounded to the nearest whole number and may not sum to 100%.

Descriptive statistics for all continuous variables used in model development are summarized in Table 2. The Shapiro-Wilk test confirmed that none of the variables followed a normal distribution (*P* < .001 for all).

**Table 2:** Descriptive Statistics and Normality Test Results of Parameters Used in the Model Development^a^

Parameter	n	Median	25th Percentile	75th Percentile	Shapiro-Wilk
**Age, y**	1709	42	35	55	0.98*
**CSF total protein, g/L**	1709	43.4	31.4	93.8	0.23*
**CSF IgG, g/L**	1709	32.92	20.45	54.65	0.61*
**CSF albumin, g/L**	1709	189	138	308	0.10*
**CSF glucose, g/dL**	1709	65.8	59.18	85.3	0.87*
**CSF sodium, mmol/L**	1709	146.2	143	148.7	0.82*
**CSF potassium, mmol/L**	1709	3.0	3.0	3.1	0.42*
**CSF chloride, mmol/L**	1709	128.1	125.9	130	0.89*
**CSF lactic acid, mmol/L**	1709	1.5	1.5	1.5	0.11*
**CSF microalbumin, g/L**	1709	209.5	189.02	218.61	0.6*
**CSF WBC, /μL**	1709	0	0	10	0.1*
**CSF RBC, /μL**	1709	10.0	0	100	0.31*
**Serum albumin, g/L**	1709	32.92	20.45	54.65	0.91*
**Serum IgG, g/L**	1709	8800	5813	11 000	0.97*
**CRP, g/L**	1709	0.6	0.2	2.6	0.12*
**ESR, mm/h**	1709	8	6	10.0	0.57*

Abbreviations: CRP, C-reactive protein; CSF, cerebrospinal fluid; ESR, erythrocyte sedimentation rate; IgG, immunoglobulin G; RBC, red blood cell; WBC, white blood cell.

a Values are given as medians (25th-75th percentiles). Normality was assessed using the Shapiro-Wilk test.

* Indicates statistical significance at *P* < .001.

### Baseline performance of the IgG index

As a baseline for comparison, the diagnostic performance of the conventional IgG index was evaluated on the independent hold-out test set. As a continuous variable, the IgG index achieved a ROC-AUC of 0.795 (95% CI, 0.740-0.844). When assessed at the established cutoff of 0.70, it demonstrated an accuracy of 76%, a sensitivity of 65%, and a specificity of 85%. Detailed performance metrics and the confusion matrix for this cutoff on the test set are presented in Table 3.

**Table 3:** Diagnostic Performance of the IgG Index at the Standard Cutoff of 0.7 for Predicting OCB Positivity^a^

OCB status^b^	Normal IgG index, No. (%)	Elevated IgG index, No. (%)	Total No.
**Negative**	168 (85)	30 (15)	198
**Positive**	51 (35)	93 (95)	144
**Total**	219	123	342

Abbreviations: IgG, immunoglobulin G; OCB, oligoclonal band.

a Confusion matrix values are derived from the hold-out test set. Results are from the independent hold-out test set, which comprised 198 OCB-negative and 144 OCB-positive cases.

b OCB status was determined by isoelectric focusing. The IgG index was classified as normal (<0.7) or elevated (≥0.7). Diagnostic performance metrics were calculated using the 0.7 cutoff for IgG index. Diagnostic metrics at this cutoff were as follows: sensitivity, 65%; specificity, 85%; positive predictive value, 76%; negative predictive value, 77%; and accuracy, 76%.

## PHASE 1: ALGORITHM SCREENING AND FEATURE IDENTIFICATION

### Initial model performance

To identify the most promising model architecture, 6 distinct ML models were evaluated on the full 24-feature set using 5-fold cross-validation. The performance of each model, reported with 95% CIs, is shown in Table 4. Gradient boosting models and random forest demonstrated superior and statistically similar discriminative ability. The top 4 performers were CatBoost (ROC-AUC = 0.939), random forest (ROC-AUC = 0.936), LightGBM (ROC-AUC = 0.935), and XGBoost (ROC-AUC = 0.933).

**Table 4:** Performance Comparison of Six Machine Learning Models Based on 5-Fold Cross-Validation^a^

Model/metric	CatBoost	Random forest	XGBoost	LightGBM	AdaBoost	Neural network
**ROC-AUC**	0.939 (0.927-0.950)	0.936 (0.925-0.947)	0.935 (0.924-0.946)	0.935 (0.924-0.946)	0.909 (0.894-0.924)	0.651 (0.625-0.677)
**PR-AUC**	0.933 (0.920-0.945)	0.929 (0.916-0.941)	0.929 (0.916-0.941)	0.930 (0.917-0.942)	0.883 (0.857-0.909)	0.632 (0.600-0.663)
**Accuracy**	0.872 (0.854-0.888)	0.860 (0.842-0.877)	0.873 (0.856-0.888)	0.868 (0.851-0.884)	0.846 (0.827-0.864)	0.643 (0.599-0.669)
**Sensitivity (recall OCB+)**	0.816 (0.771-0.871)	0.822 (0.782-0.862)	0.807 (0.767-0.855)	0.830 (0.786-0.871)	0.827 (0.770-0.869)	0.550 (0.468-0.754)
**Specificity (recall OCB−)**	0.912 (0.863-0.948)	0.888 (0.843-0.920)	0.921 (0.876-0.948)	0.895 (0.855-0.932)	0.860 (0.820-0.909)	0.711 (0.480-0.786)
**Precision (OCB+)**	0.873 (0.818-0.918)	0.843 (0.797-0.883)	0.883 (0.829-0.919)	0.854 (0.809-0.897)	0.813 (0.773-0.864)	0.586 (0.517-0.636)
**F1 score (OCB+)**	0.891 (0.874-0.907)	0.879 (0.861-0.896)	0.893 (0.875-0.907)	0.886 (0.870-0.902)	0.866 (0.847-0.884)	0.695 (0.580-0.730)
**Precision (OCB−)**	0.872 (0.845-0.903)	0.872 (0.846-0.895)	0.867 (0.845-0.894)	0.878 (0.853-0.903)	0.872 (0.845-0.903)	0.685 (0.652-0.732)

Abbreviations: AUC, area under the curve; OCB−, oligoclonal band negative; OCB+, oligoclonal band positive; PR, precision-recall; ROC, receiver operating characteristic.

a Performance metrics were calculated on the aggregated out-of-fold predictions from a 5-fold cross-validation on the development set. All values are presented as mean (95% CI). The classification thresholds for accuracy, sensitivity, specificity, precision, and F1 score were dynamically determined for each bootstrap iteration by maximizing the Youden index (J).

To formally assess whether the observed differences in ROC-AUC scores were statistically significant, pairwise comparisons were conducted using a bootstrap-based hypothesis test. The results confirmed no statistically significant differences among the top 4 models (CatBoost vs random forest, *P* = .756; CatBoost vs LightGBM, *P* = .644; and CatBoost vs XGBoost, *P* = .508). However, all top-tier models performed significantly better than AdaBoost (*P* < .05) and the MLP Neural Network (*P* < .001). These findings supported the strategic decision to construct the final specialized ensemble from different state-of-the-art implementations of the single most successful algorithm family (gradient boosting), given that performance within the top group was statistically indistinguishable.

### Feature importance analysis

To evaluate the relative contribution of each variable to model predictions, SHAP analysis was performed for the top 3 gradient boosting models.

**Figure 3:**
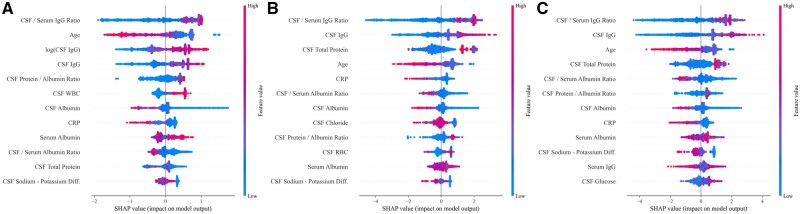
SHAP summary plots of feature importance for the top 3 machine learning models: (**A**) CatBoost, (**B**) XGBoost, and (**C**) LightGBM. Red indicates high feature values; blue indicates low values. Features are ranked by their mean absolute SHAP value. SHAP, SHapley Additive exPlanations.

For the CatBoost model (Figure 3A), the most influential features, in descending order of importance, were the CSF/serum IgG ratio, age, log (CSF IgG), CSF IgG, and the CSF protein/albumin ratio. Higher values of IgG-related features (IgG ratio, raw IgG, and log-transformed IgG) and CSF WBC count were strongly associated with an increased probability of OCB positivity. In contrast, advanced age and higher CRP levels were associated with a reduced likelihood of OCB positivity. For the XGBoost model (Figure 3B), the most prominent predictor was again the CSF/serum IgG ratio, followed by CSF IgG, CSF total protein, and age. In the LightGBM model (Figure 3C), the ranking was similarly led by the CSF/serum IgG ratio, followed by CSF IgG, age, and CSF total protein. Across all models, the directional effects were highly consistent.

### Feature selection and performance of final candidate models

To refine the feature set, a comprehensive correlation analysis using the Spearman rank correlation coefficient was performed to investigate relationships between predictor variables. The analysis identified several pairs of features with very high positive correlations, including CSF albumin and the CSF/serum albumin ratio (*r* = 0.98; *P* < .01) and CSF sodium with the CSF sodium-potassium difference (*r* = 0.99; *P* < .01). The strongest negative correlation was observed between CSF total protein and the CSF glucose/protein ratio (*r* = –0.94; *P* < .01). The complete correlation matrix is provided in Table S2.

## PHASE 2: FINAL ENSEMBLE MODEL PERFORMANCE

Based on a combined assessment of the correlation analysis and the previously conducted SHAP analysis, a final set of 8 impactful and relatively independent predictors was selected: log-transformed CSF IgG, log-transformed serum IgG, the CSF albumin/serum albumin ratio, the CSF total protein/CSF albumin ratio, the CSF glucose/CSF protein ratio, the CSF sodium-potassium difference, CRP, and patient age.

The performance of the top 3 models (CatBoost, XGBoost, and LightGBM) was then reevaluated using this refined 8-feature panel. As presented in Table 5, all 3 models maintained high discriminative power: a ROC-AUC of 0.931 (95% CI, 0.918-0.944) for CatBoost, 0.927 (95% CI, 0.914-0.940) for XGBoost, and 0.927 (95% CI, 0.912-0.940) for LightGBM.

**Table 5:** Performance Comparison of the Top 3 Candidate Models Using the Refined 8-Feature Set^a^

Model/metric	CatBoost	XGBoost	LightGBM
**ROC-AUC**	0.931 (0.918-0.944)	0.927 (0.914-0.940)	0.927 (0.912-0.940)
**PR-AUC**	0.925 (0.911-0.939)	0.921 (0.906-0.936))	0.920 (0.902-0.935)
**Accuracy**	0.856 (0.834-0.876)	0.857 (0.835-0.875)	0.860 (0.838-0.879)
**Sensitivity (recall OCB+)**	0.815 (0.740-0.888)	0.804 (0.727-0.869)	0.789 (0.739-0.851)
**Specificity (recall OCB−)**	0.886 (0.816-0.950)	0.895 (0.830-0.961)	0.913 (0.848-0.949)
**Precision (OCB+)**	0.844 (0.769-0.915)	0.852 (0.783-0.935)	0.871 (0.800-0.919)
**F1 score (OCB+)**	0.827 (0.805-0.848)	0.826 (0.802-0.848)	0.827 (0.803-0.850)
**Precision (OCB−)**	0.869 (0.830-0.911)	0.863 (0.824-0.901)	0.856 (0.826-0.891)
**F1 score (OCB−)**	0.876 (0.850-0.897)	0.878 (0.853-0.898)	0.883 (0.858-0.901)

Abbreviations: AUC, area under the curve; OCB−, oligoclonal band negative; OCB+, oligoclonal band positive; PR, precision-recall; ROC, receiver operating characteristic.

a Performance metrics were calculated for the machine learning models on the test set at 3 distinct classification thresholds. All values are presented as mean (95% CI).

**Figure 4:**
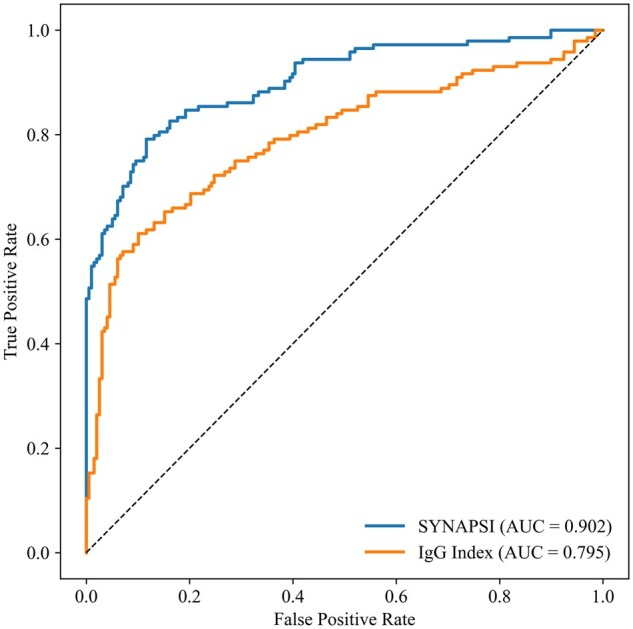
Comparative receiver operating characteristic curve analysis on the hold-out test set. The figure displays the ROC curves comparing the discriminative performance of the final SYNAPSI ensemble model (blue line; AUC = 0.902) and the conventional IgG index (orange line; AUC = 0.795). Both methods were evaluated on the same independent, sequestered hold-out test set. The SYNAPSI model demonstrated a statistically significantly superior ability to discriminate between OCB-positive and OCB-negative cases (*P* < .001 for the difference in AUC). The dashed line represents the performance of a random classifier (AUC = 0.5). AUC, area under the curve; ROC, receiver operating characteristic; SYNAPSI, Synthesis Analysis Prediction System for IgG.

The final ensemble model, named SYNAPSI, was evaluated on the independent hold-out test set. It demonstrated excellent discriminative performance, achieving a ROC-AUC of 0.902 (95% CI, 0.866-0.933). This was statistically significantly superior to the conventional IgG index, which yielded a ROC-AUC of 0.795 (95% CI, 0.740-0.844) on the same test set (*P* < .001) (Figure 4). The PR-AUC for the SYNAPSI model on the hold-out test set was 0.898 (95% CI, 0.860-0.929).

**Figure 5:**
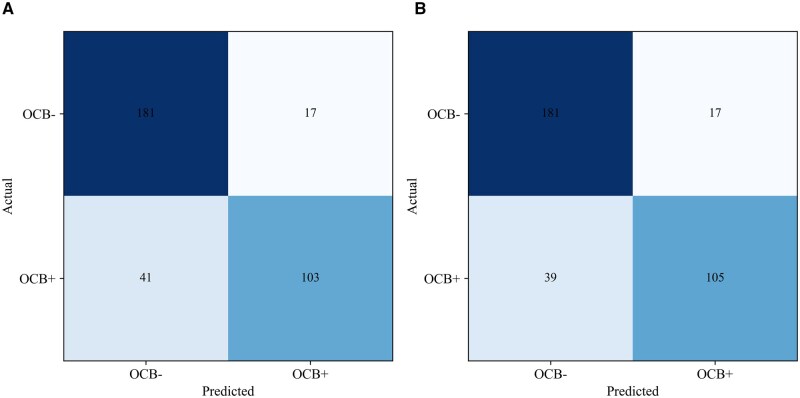
Confusion matrices for the SYNAPSI ensemble model on the hold-out test set. **A**, Performance at the optimal threshold of 0.503, determined using the Youden index (J) and F1 score. **B**, Performance at the conventional 0.5 threshold. OCB, oligoclonal band; SYNAPSI, Synthesis Analysis Prediction System for IgG.

Performance at the predetermined stable optimal threshold and the conventional 0.5 threshold is presented in Table 6. At the optimal threshold of 0.503, derived from both the Youden index (J) and F1 score, the model achieved an accuracy of 0.830 (95% CI, 0.789-0.868), a sensitivity of 0.714 (95% CI, 0.642-0.782), and a specificity of 0.916 (95% CI, 0.877-0.953). The confusion matrix for this threshold indicated 103 true positives and 181 true negatives (Figure 5A). For comparison, the conventional 0.5 threshold resulted in a slightly higher accuracy (0.836 vs 0.830) and sensitivity (0.728 vs 0.714). Furthermore, the SYNAPSI model was well calibrated, as the predicted probabilities closely aligned with the observed frequencies across the probability range, demonstrated by the reliability curve (Figure S2).

**Table 6:** Performance Metrics of the SYNAPSI Model at 3 Different Classification Thresholds on the Independent Test Set^a^

Metric	F1-optimal and Youden threshold	Default 0.5 threshold
**Threshold-independent performance**	0.902 (0.866-0.929) 0.898 (0.860-0.929)	
** ROC-AUC**		
** PR-AUC**		
**Threshold-dependent performance**		
** Accuracy**	0.830 (0.789-0.868)	0.836 (0.795-0.874)
** Sensitivity (OCB+)**	0.714 (0.642-0.782)	0.728 (0.659-0.796)
** Specificity (OCB−)**	0.916 (0.877-0.953)	0.916 (0.877-0.953)
** Precision (OCB+)**	0.860 (0.797-0.922)	0.863 (0.800-0.924)
** Precision (OCB−)**	0.814 (0.760-0.865)	0.822 (0.770-0.871)
** F1 score (OCB+)**	0.780 (0.724-0.831)	0.789 (0.734-0.841)
** F1 score (OCB−)**	0.862 (0.825-0.894)	0.866 (0.829-0.899)

Abbreviations: AUC, area under the curve; OCB−, oligoclonal band negative; OCB+, oligoclonal band positive; PR, precision-recall; ROC, receiver operating characteristic.

a Performance metrics were calculated on the independent hold-out test set. All values are presented as mean (95% CI).

### Validation in an independent cohort

The final SYNAPSI model was tested on an independent external cohort of 49 samples that had not been used at any prior stage. The OCB pattern distribution for this cohort was as follows: type 1 (n = 4, 8%), type 2 (n = 9, 18%), type 3 (n = 16, 33%), type 4 (n = 20, 41%), and type 5 (n = 0, 0%). In this cohort, the model demonstrated robust performance, achieving an accuracy of 90%, a sensitivity of 96%, and a specificity of 83%. The detailed confusion matrix and full performance metrics for the external validation are presented in Table 7.

**Table 7:** Performance of the SYNAPSI Model on the Independent External Validation Cohort (n = 49)

Characteristic/metric	Value
**Cohort composition, No. (%)**	
** OCB-positive cases**	25 (51)
** OCB-negative cases**	24 (49)
**Confusion matrix results, No.**	
** True positives**	24
** True negatives**	20
** False positives**	4
** False negatives**	1
**Calculated performance metrics, %**	
** Accuracy**	90
** Sensitivity (recall OCB+)**	96
** Specificity (recall OCB−)**	83
** Precision (OCB+)**	86
** Precision (OCB−)**	95

Abbreviations: OCB−, oligoclonal band negative; OCB+, oligoclonal band positive; SYNAPSI, Synthesis Analysis Prediction System for IgG.

## DISCUSSION

### Summary of principal findings

In this study, we developed an ML-based model, SYNAPSI, that utilizes CSF and serum parameters to accurately predict OCB positivity. The final ensemble model significantly outperformed the conventional IgG index. In our study, the IgG index yielded a sensitivity of 65%, a specificity of 85%, and a ROC-AUC of 0.795 (95% CI, 0.740-0.844) on the hold-out test set, aligning with prior findings indicating that this marker may fail to identify a substantial portion of OCB-positive cases. This reinforces the need for improved predictive strategies that integrate routinely available laboratory markers into a unified framework.

By comparison, our initial screening of multiple algorithms identified gradient boosting machines as the most promising candidates. As a result, our final model was constructed as a weighted ensemble of the top 3 performers, XGBoost, LightGBM, and CatBoost. The rationale for this ensemble approach is that while each algorithm is individually powerful, they employ distinct methods for tree construction and data handling. Combining them often yields a more robust and generalizable model, mitigating the specific biases of any single algorithm.

This final SYNAPSI model demonstrated strong discriminative ability on a hold-out test set, achieving a high ROC-AUC of 0.902 (95% CI, 0.866-0.933). To better contextualize the performance of SYNAPSI, we compared it with conventional methods and relevant literature.

### Comparison with existing methods and literature

Our model addresses a well-recognized gap in laboratory medicine, where clinicians often face a trade-off between the high sensitivity of IEF, which is labor-intensive and subjective, and the rapid yet less sensitive IgG index.^10‐12^ In our study cohort, the IgG index, consistent with prior reports, failed to detect a substantial proportion of OCB-positive cases.^10‐13^ Nevertheless, the IgG index remains a valuable quantitative tool, providing an objective measure that can be particularly useful in preventing misdiagnosis in atypical cases or in settings where the more complex IEF is unavailable.^13^ The superior performance of the SYNAPSI model suggests that a data-driven integration of multiple routine laboratory parameters can produce a more reliable and discriminative predictive signal than any single calculated index, a finding that is consistent with the growing body of literature on the utility of ML in laboratory medicine.^9^^,^^14^

In the literature, OCB positivity in CSF is reported in approximately 85% to 95% of patients with MS. A negative OCB result in a clinically suspected MS case often necessitates reevaluation of the diagnosis.^15^^,^^16^ Despite its rapid turnaround and broad availability, the IgG index has been criticized for its low sensitivity and limited diagnostic accuracy.^13^^,^^16^^,^^17^ Multiple studies have shown that the IgG index fails to match the diagnostic performance of OCB testing, particularly in terms of sensitivity.^10^^,^^18^ Reports also exist of cases in which the IgG index is within the normal range despite positive OCB results and vice versa.^10^^,^^16^ Additionally, nonlinear approaches such as the Reiber formula have been proposed as potentially superior alternatives to the linear IgG index.^16^

Our approach, which relies on routinely available biochemical markers, complements other recent efforts that have utilized more specialized research-grade assays to investigate the underlying pathophysiology of OCB status. While these findings underline the limitations of current routine markers, several recent studies have explored alternative approaches. For instance, a recent study by Gaetani et al^19^ used a proximity extension assay to profile 92 immune-related proteins in the CSF of patients with MS. They successfully identified a distinct panel of biomarkers (including IL-12B, CD5, and CX3CL1) capable of distinguishing OCB-positive MS from controls with high accuracy. Unlike these research-grade assays, our approach focuses entirely on standard, readily available laboratory tests. While such studies provide invaluable insights into the distinct immunopathologic signatures associated with OCB status, their reliance on highly specialized and less accessible analytical platforms limits their immediate applicability in routine clinical laboratories. In contrast, the SYNAPSI model was deliberately developed using only standard, universally available laboratory tests, positioning it as a pragmatic, immediately deployable tool to enhance diagnostic efficiency and objectivity within current laboratory workflows, rather than as a platform for novel biomarker discovery.

Our study’s approach of predicting the OCB result itself, rather than using it as a predictor, distinguishes it from other models in the literature. Other modeling efforts have taken a different direction, as illustrated by Chen et al,^20^ who developed a logistic regression model to differentiate MS from other neurologic diseases, incorporating OCB status as one of its key input features. Their model achieved a high AUC of 0.916 and demonstrated the value of combining OCB results with other biochemical markers. However, their work focuses on the clinical interpretation following the performance of an OCB test. In contrast, our SYNAPSI model is designed to address a more foundational challenge within the laboratory workflow: predicting the OCB result before or in place of the cumbersome IEF test. By leveraging more advanced, nonlinear ML architectures and a broader feature set, our model provides a tool aimed at directly augmenting the diagnostic testing process itself, rather than the subsequent clinical differentiation. Such findings collectively highlight the ongoing search for practical, high-performance tools in laboratory medicine.

After establishing the comparative performance advantages of the SYNAPSI model, it was necessary to define how these results could be operationalized through an optimal probability threshold.

### Selection of an optimal decision threshold

While the ROC-AUC provides a global measure of a model’s discriminative ability, its clinical translation requires selecting a decision threshold to convert probability scores into a binary classification (OCB-positive or OCB-negative). The conventional 0.5 threshold is often suboptimal because it assumes equal costs for false positives and false negatives. To make the model actionable in practice, an evidence-based probability cutoff is required. Therefore, to identify a more clinically relevant cutoff point, we employed 2 established optimization strategies on the development set.

The first approach maximized the F1 score, which represents the harmonic mean of precision (positive predictive value) and sensitivity (recall). This method seeks an optimal balance between correctly identifying true positives and minimizing false-positive results. In parallel, we maximized the Youden index (J) to optimize the sensitivity-specificity trade-off. This method aims to maximize the overall diagnostic accuracy across both positive and negative classes. This convergence reinforces the robustness of the identified threshold, supporting its adoption as a data-driven standard rather than an arbitrary choice. Both methods converged on 0.503. Adopting this data-driven threshold, rather than an arbitrary default, ensures that the SYNAPSI model’s performance is optimized for the specific context of OCB classification, prioritizing a balanced diagnostic performance. In clinical practice, lowering it toward 0.50 can increase sensitivity at the expense of more false positives.

Following the selection of the optimal decision thresholds, we explored the factors driving the model’s predictions through feature importance analysis.

### Interpretation of feature importance

Given the clinical imperative for early detection, the SYNAPSI model was implemented with a 0.503 threshold—the median of the F1-optimal and Youden J thresholds—selected to achieve balanced sensitivity and specificity. In clinical applications, sensitivity can be further increased by lowering the threshold to 0.50, at the expense of specificity.

To understand the factors driving the model’s predictions, we performed SHAP analysis, which identified the most influential predictors. Across all high-performing models, CSF IgG-related parameters, particularly the IgG index and raw CSF IgG levels, were consistently ranked as the most important features. This is clinically and pathologically congruent, as the presence of OCBs is defined by the intrathecal synthesis of IgG. It is noteworthy that while the log-transformed CSF IgG feature was also highly predictive, it did not always appear in the top-ranked features for every model. This is an expected outcome due to multicollinearity between the highly correlated CSF IgG, log-transformed CSF IgG, and IgG index features. Tree-based models typically select 1 representative feature from highly correlated sets; once chosen, the apparent importance of the others diminishes. This observation does not detract from the value of the log-transformed feature but rather highlights the nuanced decision-making processes of the different algorithms.

The model’s biological plausibility is supported by prior literature. Elevated CSF IgG reflects local B-cell activation and antibody synthesis, both closely associated with OCB positivity.^21^ The CSF/serum albumin ratio (albumin index) serves as a marker of blood-brain barrier integrity, while total CSF protein levels reflect inflammation and axonal damage.^22^^,^^23^ The SHAP analysis revealed that features such as CSF IgG, albumin ratios, and electrolyte differences made substantial contributions to the model’s output. However, it is important to emphasize that SHAP values reflect the model’s internal feature importance and do not imply clinical or statistical causality. Further biological and clinical studies are necessary to validate the mechanistic significance of these findings.

The model also successfully integrated signals from other routine parameters. Patient age was a significant predictor, with younger age being associated with OCB positivity, consistent with the typical onset age of MS and a more active immune response in younger patients.^24^^,^^25^ Systemic inflammatory markers like CRP were also influential; elevated CRP levels, indicating systemic rather than CNS-localized inflammation, correctly shifted the model’s prediction away from OCB positivity.^26^ The model’s reliance on the CSF sodium-potassium difference as a key discriminator, while subtle, may reflect the underlying pathophysiology of neuroinflammation. While significant changes in CSF electrolytes are not typically expected in chronic inflammatory states such as MS, recent studies suggest that minor variations can be associated with disease activity. The tight homeostatic control of CSF ion composition can be influenced by intrathecal inflammation through various mechanisms. For instance, inflammatory mediators present in the CSF of patients with MS can alter the function of ion channels and transporters on neural and glial cells, potentially leading to subtle shifts in sodium and potassium concentrations. Furthermore, the Na-K-2Cl cotransporter, which is crucial for CSF secretion and brain potassium homeostasis, could be dysfunctional during inflammation, contributing to altered ion gradients. This is supported by findings that link other electrolytes, such as CSF chloride, to disease activity in MS, suggesting a broader impact of neuroinflammation on CSF ion composition.^27‐29^ The importance of potassium homeostasis is further highlighted by studies on the astroglial potassium channel Kir4.1, against which autoantibodies have been detected in a subset of patients with MS.^30^ Disruption of this channel can impair potassium balance and contribute to the pathological state. Therefore, the CSF sodium-potassium difference may serve as a faint but detectable signal of the complex immunologic and metabolic disruptions associated with the high intrathecal immune activity characteristic of OCB positivity. Our SHAP analysis, which showed that a decreasing sodium-potassium difference increased the likelihood of predicting OCB positivity, appears consistent with these findings in the literature. This ability to synthesize information from multiple, diverse parameters underscores a key advantage of ML over single-marker methods.

### Clinical utility and implications for laboratory medicine

While the sensitivity of 71% at the optimal threshold indicates that SYNAPSI is not a replacement for the gold-standard IEF, its clinical value should be assessed holistically. The model’s primary advantage lies in its superior overall discriminative power (ROC-AUC = 0.902), which integrates multiple parameters into a single, objective probability score. Crucially, its high specificity of 92% (compared to 85% for the IgG index) can significantly reduce the rate of false-positive predictions, minimizing unnecessary clinical anxiety and follow-up investigations in low-probability cases. Therefore, the intended use case for SYNAPSI is not to replace IEF but to act as an intelligent decision support and workflow optimization tool. For instance, a high probability score generated by the model could flag a sample for prioritized IEF analysis, potentially shortening the diagnostic timeline for cases with a high pretest likelihood of MS. Conversely, a very low probability score could provide clinicians with an early, data-driven indication of a low likelihood of intrathecal synthesis, helping to manage the diagnostic process while awaiting the definitive IEF result.

Beyond statistical performance, SYNAPSI has the potential to offer practical benefits for laboratory workflow and clinical decision-­making. The statistical performance of the SYNAPSI model, while academically significant, finds its true value in its potential for tangible, real-world impact on laboratory operations and clinical decision-making. One of the most immediate implications is for laboratory workflow. By providing an accurate, quantitative prediction of OCB status from routine biochemical tests, SYNAPSI could function as an intelligent decision support tool. This could be used, for example, to alert clinicians to a high probability of OCB positivity long before the IEF results are available or to add confidence in cases where the IEF banding pattern is ambiguous or difficult to interpret. This optimization would enable highly skilled laboratory technologists to redirect their focus toward the most complex cases, thereby improving the overall efficiency of the diagnostic process.

Beyond workflow, SYNAPSI could serve as a powerful tool that augments, rather than replaces, the clinician’s and clinical pathologist’s judgment. By providing an objective risk score based on the integration of multiple parameters, the model can complement expert interpretation. This is particularly valuable in diagnostically challenging cases, where an objective data point can help increase diagnostic confidence, reduce interobserver variability, and guide the diagnostic process more effectively.

The reliability of this tool is further underscored by its strong calibration (Figure S2), which ensures that the predicted probability scores are trustworthy representations of the true likelihood of OCB positivity. This is a critical feature for any model intended to support clinical decision-making, as it provides clinicians with a risk estimate they can confidently interpret.

An exploratory analysis of the misclassified cases in the hold-out test set (Figure 5) provided further insights into the model’s behavior. The 41 false-negative cases (true OCB-positive cases classified as negative) represented a diagnostically challenging subgroup with a median IgG index of 0.61 (mean = 0.70), well below the conventional cutoff. This suggests that in these cases, intrathecal IgG synthesis was not strongly reflected in bulk quantitative markers, making them difficult to detect for both the conventional index and our ML model. Conversely, an examination of the 17 false-positive cases revealed a common characteristic: a notable proportion of these cases had IgG index values that were borderline high (median = 0.69; mean = 0.90). This indicates that the model was often correctly identifying a quantitative signal of intrathecal synthesis, which, in these specific cases, was below the threshold for a positive result by the qualitative gold-standard IEF method.

While a formal health-economic analysis is beyond the scope of the present study, the implementation of SYNAPSI offers the potential for significant resource optimization. Given that the reagent and labor costs of an IEF test are substantially higher than the combined cost of the routine biochemical markers used by the model, even a modest reduction in the number of IEFs performed could lead to considerable cost savings. This could be achieved through more efficient workflows, such as prioritizing IEF testing for high-probability samples. Such optimization would also free up skilled technologist time for more complex tasks. A formal cost-effectiveness study, weighing these potential gains against the costs of model development and maintenance, would be a valuable future research direction to fully elucidate the model’s economic impact.

### Framework for implementation: Integrating SYNAPSI into the clinical workflow

A validated algorithm is merely the first step; bridging the well-documented “implementation gap” between a research model and a clinically integrated tool requires a structured approach. The successful deployment of SYNAPSI necessitates moving beyond its current proof-of-concept stage. As SYNAPSI is intended to guide clinical reporting, its deployment within a single health system would likely proceed as a laboratory developed test, requiring stringent local performance evaluation. A significant technical barrier is the integration of the model with existing laboratory information systems (LIS). Overcoming this requires a dedicated, collaborative effort involving pathologists, data scientists, and institutional information technology specialists to develop seamless data pipelines and ensure the model’s output is integrated effectively into the postanalytical workflow.

However, the most critical determinant of successful ML adoption is the trust and acceptance of its end users—clinical pathologists and clinicians. Trust is not built on performance metrics alone; it is cultivated through model transparency. Our use of SHAP analysis to provide clear explanations for the model’s predictions is a crucial step in building this trust, as it allows users to understand and verify the model’s logic. This deliberate, user-centric approach is essential to ensure that SYNAPSI is not just a validated algorithm but a valued and effective clinical tool.

Long-term maintenance is critical for any clinically implemented ML model. To ensure sustained performance and evaluate for model drift over time, a monitoring strategy would be essential. This would involve periodic revalidation of the model on prospectively collected data to confirm its performance metrics, particularly its ROC-AUC and calibration. Furthermore, tracking the statistical distributions of both the model’s output probabilities and the key input features would serve as an early warning system for shifts in patient populations or analytical methods. Regarding broader implementation, it is important to note that the SYNAPSI model is built upon the biochemical parameters required for the IgG index calculation. Therefore, its use is intended for centers that already perform these fundamental tests, as it leverages these existing data to provide a superior predictive signal.

### Methodological strengths and study limitations

A primary strength of this study is its foundation upon a large, well-curated clinical data set from a single tertiary care center, which ensures data consistency due to standardized laboratory protocols. The methodological rigor was further enhanced by the strict partitioning of data into a development set and a blinded hold-out test set. This approach ensures an unbiased assessment of the model’s ability to generalize to new, unseen data. Furthermore, the performance on an independent external validation cohort suggests the model is robust and not overfit to our institution’s specific population.

The methodological framework was designed to ensure robustness and transparency. An initial screening of 6 different algorithms confirmed the superior performance of the gradient boosting family. Although random forest also demonstrated strong and statistically similar performance (*P* > .05), the final ensemble was strategically constructed from the top 3 gradient boosting implementations (CatBoost, XGBoost, and LightGBM). This decision was based on the rationale that combining different state-of-the-art interpretations of a single, powerful methodology creates a more specialized and synergistic model. Logarithmic transformations were applied to IgG concentrations, which successfully normalized their highly skewed distributions (skewness = 5.19 for CSF IgG) and enhanced model stability.

Despite these strengths, it is imperative to acknowledge the study’s limitations. The principal limitation is its retrospective design. Second, the model was developed at a single institution, which, while ensuring data consistency, inherently limits the proven generalizability of the findings. The most significant limitation is the reduction of the data set from an initial 4346 to 1709 cases due to the exclusion of records with missing OCB status. This complete-case analysis, while methodologically chosen to prevent biases from data imputation, may limit the model’s representativeness.

Additionally, the external validation cohort, while independent, was of modest size (n = 49), which limits the statistical power of this validation step. Although the performance metrics were strong, these results should be interpreted with caution and viewed as a promising but preliminary indication of the model’s generalizability. Confirmation in larger, multicenter external cohorts is a critical next step.

### Future directions and concluding remarks

Building upon the findings and limitations of this study, the most essential next step is to conduct a large-scale, prospective, multicenter validation study. This is the gold standard for confirming a model’s performance and is critical for assessing its true generalizability across diverse patient populations and varied laboratory practices. Concurrently, future research should focus on the practicalities of LIS integration and its downstream impact on key laboratory and clinical metrics, including diagnostic turnaround time and pathologist workload.

In conclusion, this study robustly demonstrates that an ML model, using only routinely collected laboratory data, can significantly outperform the conventional IgG index for predicting OCB status. While prospective validation is an essential prerequisite for implementation, SYNAPSI represents a tangible step toward leveraging existing data and artificial intelligence to create more objective and efficient tools in laboratory medicine, ultimately aiming to both enhance diagnostic workflows and support clinical decision-making.

It should be emphasized that such tools are designed to augment and support expert interpretation as part of the diagnostic workflow, rather than to replace the definitive gold-standard methods.

## Supplementary Material

aqaf119_Supplementary_Data

## Data Availability

The data underlying this article cannot be shared publicly due to ethical and privacy restrictions related to patient information. The data will be shared on reasonable request to the corresponding author.
