# Supplementary material for: A machine learning model for predicting oligoclonal band positivity using routine cerebrospinal fluid and serum biochemical markers
Source: Am J Clin Pathol. 2025 Dec 6;164(6):933–45. doi: 10.1093/ajcp/aqaf119 (PMC12782304; doi:10.1093/ajcp/aqaf119)
Supplement: aqaf119_Supplementary_Data [file aqaf119_supplementary_data.zip › ajcp-2025-08-0422-File009.docx]

**Supplementary Table 1.** Comparison of Demographic Characteristics Between the Development and Hold-Out Test Sets.

| **Characteristic** | **Development Set (n=1,367)** | **Test Set (n=342)** | **p-value^*^** |
| --- | --- | --- | --- |
| Age (years), median [IQR] | 42 [35-55] | 42 [35-54] | 0.981 |
| Sex, n (%) |  |  | 0.945 |
| Female | 576 (42%) | 145 (42%) |  |
| Male | 791 (58%) | 197 (58%) |  |

**^*^**Continuous variables were compared using the Mann-Whitney U test; categorical variables were compared using the Chi-squared test.

**Abbreviations:** IQR, interquartile range.
